# How Do Orthopaedic Patients Prefer to Be Contacted During a Pandemic?

**DOI:** 10.7759/cureus.25049

**Published:** 2022-05-16

**Authors:** David Fellows, Jamie Hind, Gur Aziz Singh Sidhu, Veda Vani Amara, Neil Ashwood

**Affiliations:** 1 Trauma and Orthopaedics, Oxford University Hospitals NHS Foundation Trust, Oxford, GBR; 2 Trauma and Orthopaedics, University Hospitals of Derby and Burton NHS Foundation Trust, Burton-on-Trent, GBR

**Keywords:** patient’s satisfaction, elective orthopaedic surgery, survey, communication technology, communication in healthcare, covid-19 pandemic

## Abstract

Introduction

Communication with patients is a vital part of the surgical pathway, and when done effectively, it can greatly improve patient outcomes and patient satisfaction and reduce canceled appointments. Different forms of communication work well for different patient demographics, and it is important to optimize communication techniques. We designed a study to review the communication preferences of orthopedic patients during the COVID-19 pandemic.

Methods

A cross-sectional study was performed by asking patients who were due to undergo orthopedic procedures to answer a questionnaire on their communication preferences, the reminder notice period for appointments, and safety and satisfaction ratings during the COVID-19 pandemic.

Results

Communication method preferences are influenced by patient factors such as gender and age. Phone calls were the most popular communication method throughout all patient groups, with 61% selecting it as their preference. Younger patients preferred multiple communication methods of phone calls, texts, and emails, whereas the older group had a stronger preference for letters. Letters were more popular among females (28% compared to 10% of males), whereas males had a stronger preference for other communication methods. The majority of patients said they would not have liked a letter prior to their clinic appointment (65%). Of those who indicated a preferred notice period, 73% would have liked five days or less notice prior to their clinic appointment, while 65% would have liked 10-14 days notice prior to their surgery. The average safety rating was 55%. The overall satisfaction rating with the communication process was 71.7%.

Conclusion

The COVID-19 pandemic has changed patient feelings towards healthcare and, as a result, changed the way healthcare is delivered. Communication method preferences among trauma and orthopedic patients vary and depend on factors such as gender and age. If healthcare departments can optimize their communication processes, they will improve their patient outcomes and enhance their resources.

## Introduction

The benefits of effective communication between healthcare providers and surgical patients have been well documented and researched. Better communication leads to a good relationship with the patient and has been shown to improve patient autonomy [1,2], patient satisfaction [3], fewer canceled clinic or surgery appointments [4,5], and overall improved outcomes for patients [6]. For patients undergoing surgery, communication in the pre-operative period is a vital part of the surgical process. Good communication with patients leads to improved outcomes as a result of better patient understanding of their surgeries and improved compliance with planning and optimization pre-operatively [7-9].

The COVID-19 pandemic has affected the way patients feel about healthcare and their overall safety when attending routine appointments or for planned treatments. Changes in patient feelings towards their healthcare throughout the pandemic have been well documented already, with many feeling more worried about receiving healthcare and that they are less safe [10,11].

This study aimed to look at how patient behaviors and feelings have changed during the pandemic, both regarding their communication preferences and their overall feeling of safety when attending elective hospital appointments. It is a follow on to a recent study by Ghunimat et al., performed before the pandemic, which found that among patients undergoing elective orthopedic surgery, those aged over 65 preferred traditional letters as a form of communication, whereas younger patients had a range of preferences between text, email, and phone calls [12].

The pandemic has greatly changed the way healthcare is planned and delivered, and during a time when remote working and planning have become the norm, communication with patients is more important than ever.

## Materials and methods

A cross-sectional** **study was performed using a questionnaire answered by 111 patients due to undergo elective orthopedic procedures during the COVID-19 pandemic at an NHS hospital in the United Kingdom.

Study design

The questionnaire was developed within the trauma and orthopedic department using a focus group, which included both staff and patients, and was trialed on 10 patients initially. The questionnaire was designed using established principles such as a small number of clear, high-quality research questions that are relatively quick to answer and provide reliable results which measure the outcomes being looked at [13].

The questionnaire included nine questions (see Appendix). The questionnaire asked about communication method preferences, which included phone calls, text messages, e-mails, and letters. Patients were able to select more than one communication method, and they were also asked to indicate which method they preferred.

The questionnaire also asked how many days of notice patients prefer before attending their appointment and before their surgery, whether they would like confirmation by letter for their appointment, overall satisfaction with the communication up to that point, and how safe the patient felt at their appointment, and would they attend a clinic appointment again in the current pandemic.

This study was done in the frame of an audit done at a hospital in the University Hospitals Derby and Burton NHS Foundation Trust, UK. It was part of service evaluation and did not meet the criteria for research, therefore did not require further ethical approval. It was registered as a local audit, number 3679.

Verbal consent was obtained by each patient who undertook the survey for their responses to be used for research. No identifiable patient information was captured or used.

Data collection

All patients, including both adult and pediatric patients, who attended outpatient orthopedic clinic appointments were asked to complete the questionnaire.

The questionnaire responses were collected over two months, from October 2020 to December 2020, during the second wave of the COVID-19 pandemic in the UK. Patients were given the questionnaire at their appointment and asked to fill it out and hand it back to the clinical team before they left. If the patient had an accompanying parent or guardian, for example, pediatric patients, then the patient or guardian completed the questionnaire with the patient.

In total, 132 questionnaires were given out; 111 were completed and used in this study. Three questionnaires were discarded as they were incomplete, 10 patients declined to complete the questionnaire, and eight patients were not given the questionnaire due to cognitive issues and their carer not being fully aware of the patient's injury. This gave a response rate of 84%.

Data analysis 

The questionnaire responses were analyzed, and responses for the questions relating to communication preference were given a weighting based on the number of choices each patient made. The choices were further looked at by gender of the patients and then by the age of the patients to compare how different patient groups preferred each communication method. T-test statistical analysis was used to look at variables between different patient groups from this study, where appropriate.

## Results

Communication preferences

**Figure 1:**
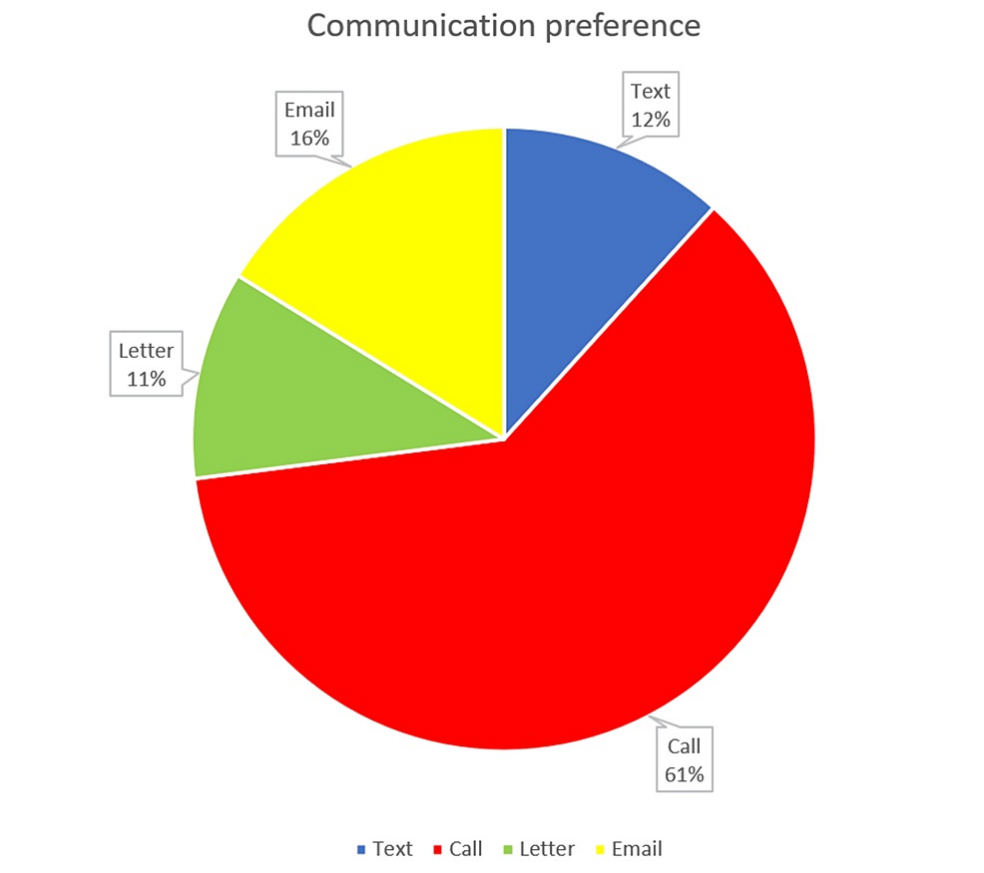
Pie chart showing overall favored communication preference among all patients

When asked to state which communication method was preferred, phone calls were the overwhelming favorite (68; 61%), followed by email (18; 16%), then text (13; 12%), and then letters (12; 11%; see Figure 1).

**Figure 2:**
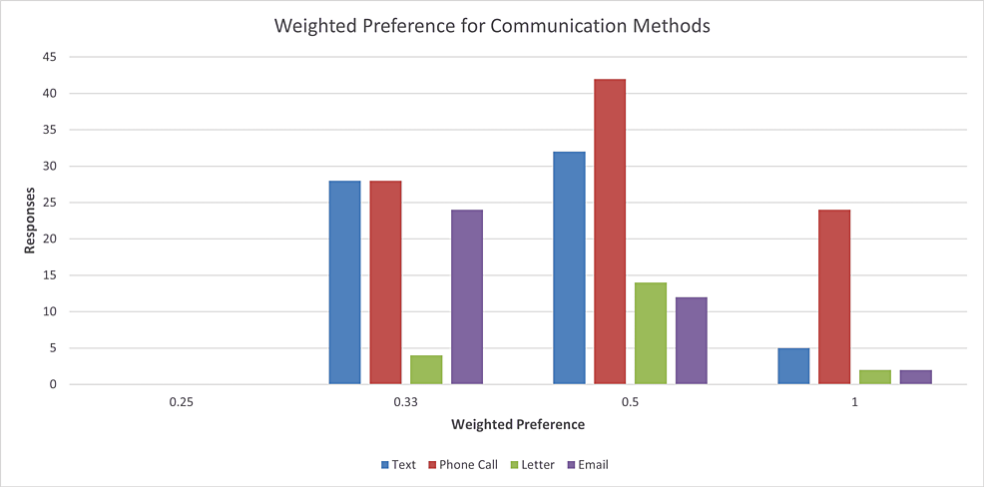
Bar chart showing weighted preferences for each communication method The X-axis shows the weighting of each choice (patients choosing all four choices gave options a weighting of 0.25, three choices weighting of 0.33, two choices weighting of 0.5, and one choice weighting of 1). The Y-axis shows a number of responses.

When asked to indicate all communication methods that they are happy with, again, phone calls came out on top with 98 (88.3%) choosing those, followed by 68 (61.3%) people choosing text messages. Emails were the third most popular, with 38 (34.2%) choosing those, followed by 20 (18%) selecting letters. This shows that often patients who prefer phone calls are also happy to receive text messages and, to a lesser extent, emails. Communication via letters remains low, with a small proportion of patients content with this form of communication. No patients chose all four communication methods together (Figure 2).

**Figure 3:**
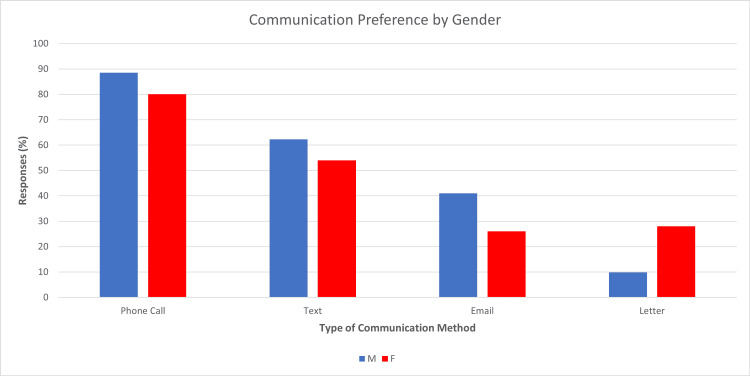
Bar chart of preferences for males (blue) and females (red) for each communication method A number of responses is shown as a percentage of each gender (Y-axis) vs. type of communication method (X-axis).

A total of 50 females and 61 males completed the questionnaires. Comparing between genders, males preferred phone calls more, with 54 (89%) choosing this option, compared to 40 (80%) female respondents. Males also showed a stronger preference for text messages, with 38 (62%) males compared to 27 (54%) females choosing this option, and emails showed similar results, with 25 (41%) males choosing this option compared to 13 (26%) females. Letters were more popular among females, with 14 (28%) choosing this option compared to six (10%) males (Figure 3).

**Figure 4:**
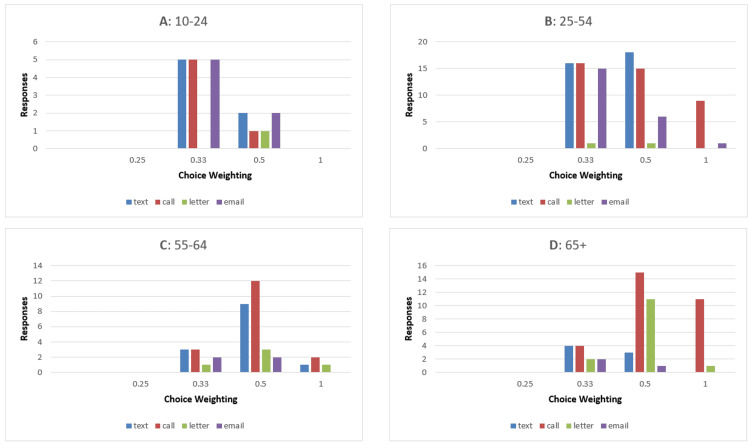
Bar charts showing weighted options for communication preferences by age groups The X-axis shows the weighting of each choice (patients choosing all four choices gave options a weighting of 0.25, three choices weighting of 0.33, two choices weighting of 0.5, and one choice weighting of 1), the Y-axis shows a number of responses. A: 10-24-year-olds; B: 25-54-year-olds; C: 55-64-year-olds; D: over 65-year-olds

There is a discrepancy in communication preferences between different age groups (Figure 4). When responders are broken down by age, 13 are aged 10-24, 47 aged 25-54, 20 aged 55-64, and 31 aged 65 and over. The most popular communication methods for 10-14 year-olds were text and email, with 54% of this age group selecting these options, closely followed by phone calls with 46% choosing this option, and letters were the least popular with 8% picking this option (Figure 4A).

Responders aged 25-54 showed a similar theme to the youngest age group. Phone calls were the most popular, with 85% of this age group choosing this option, followed by text messages with 72%. Emails were chosen by 47%, while letters were again the least popular with 4% of responders (Figure 4B).

In the 55-64 age group, phone calls and texts remained high, with 85% and 65% indicating these options. The main difference between this age group and the younger age group was that there was more of a preference for letters and less of a preference for emails, with 25% and 20% picking these options, respectively (Figure 4C).

The 65+ age group shows a big increase in preference for letters in comparison to the other groups, with 45% selecting this form of communication. Phone calls were the overwhelming favorite in this age group, with 97% opting for this method, while text messages and emails were less popular, with 23% and 10%, respectively (Figure 4D).

Reminders

**Figure 5:**
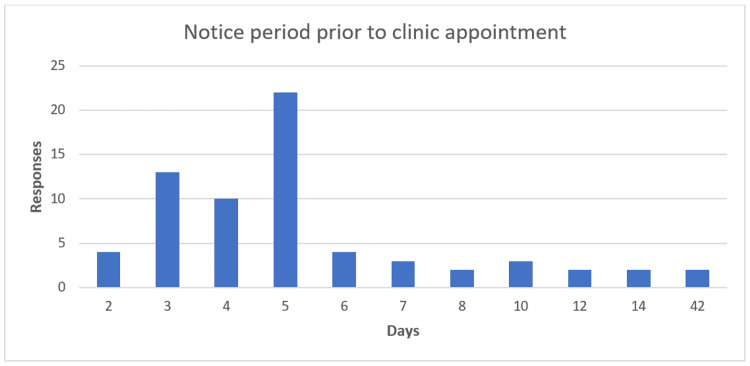
Bar chart showing patient preferred notice period prior to a clinic appointment The X-axis shows the number of days; Y-axis shows the number of responses.

Patients were asked whether they would have liked a confirmatory letter before their pre-operative appointment. The majority of patients (65%) indicated that they did not want a confirmatory letter prior to their appointment. Thirty-nine (35%) said they would have liked a letter. When asked when they would like to be given a reminder before their appointment, 67 patients answered. The most common response from 33% of patients was to receive a reminder five days before the appointment, with 73% of responses indicating a reminder within five days prior to the appointment. The mean number of days of notice was 6.3; the median was five days. The responses went up to 42 days' notice (Figure 5).

**Figure 6:**
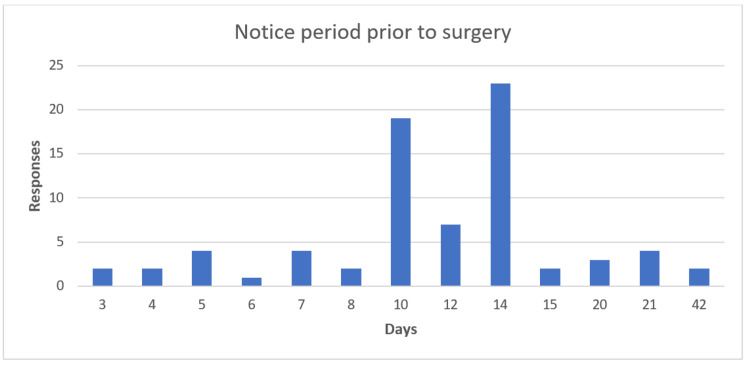
Bar chart showing patient preferred notice period prior to surgery The X-axis shows the number of days; Y-axis shows the number of responses.

When asked about a notice period prior to surgery, 75 patients answered this question. This time the responses indicated a longer period of time, with the most common responses being 10 days (25%), 12 days (9%), and 14 days (31%). The mean number of days' notice was 11.5; the median was 12. Again, the responses went up to 42 days prior to the appointment (Figure 6).

Safety and satisfaction

**Figure 7:**
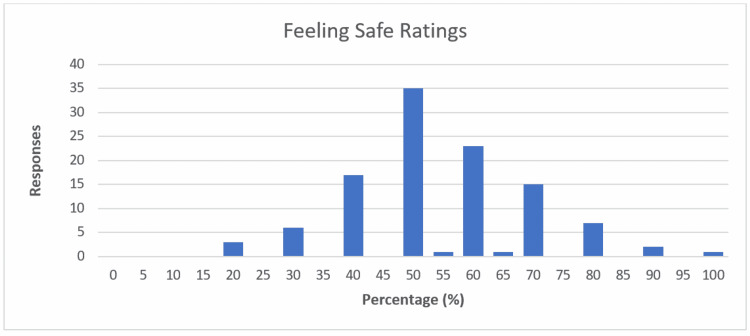
Bar chart showing patients' feeling safe ratings The X-axis shows the percentage of satisfaction; Y-axis shows the number of responses.

Patients were asked to rate how safe they felt with undergoing pre-operative appointments and surgery during the COVID-19 pandemic as a percentage. The average safety rating was 55% (see Figure 7). When separated into groups based on whether or not patients were out of the house during the day at work or school, 71 patients who worked away from home or attended school had an average safety rating of 57.5%, while 40 patients who were retired, unemployed, or working from home had an average safety rating of 49.5%. This was statistically significant (p=0.007). 

When looking at average safety ratings by patients separated into groups by age, patients aged <25 was 60.2%, 25-54-year-olds was 54.0%, 55-64-year-olds was 60.3%, and >64-year-olds was 49.4%. These differences were not statistically significant (p=0.34).

**Figure 8:**
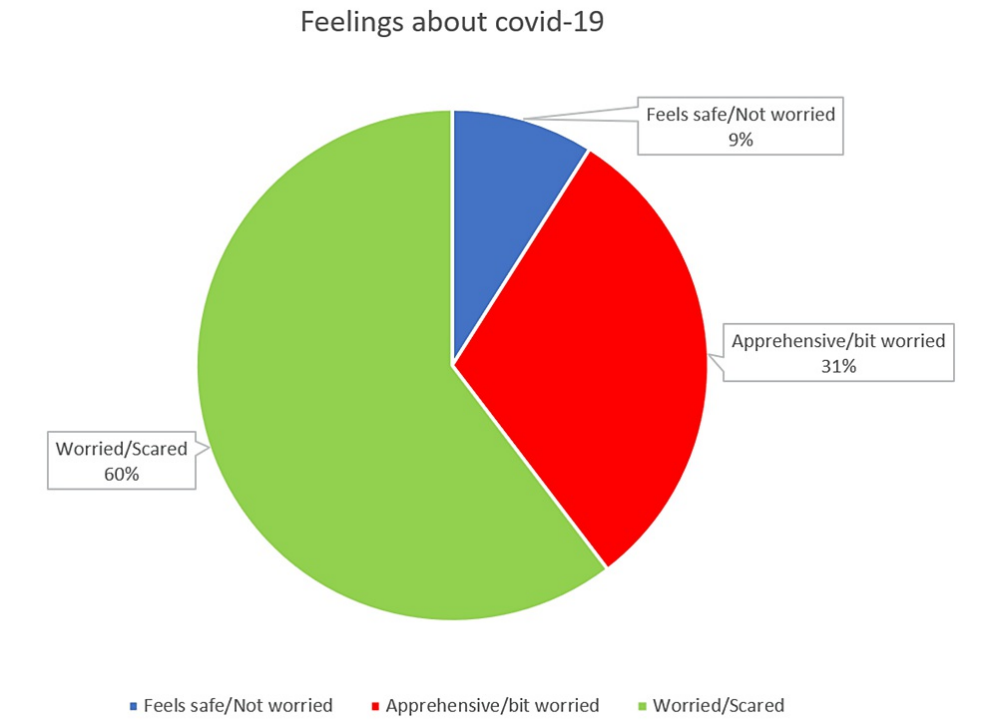
Pie chart showing the percentage of patients feeling safe/not worried; apprehensive/bit worried; and worried/scared

Patients were asked to provide specific comments regarding their feelings toward the COVID-19 pandemic. Most patients (67; 60%) said they felt worried or scared, 34 (31%) said they felt apprehensive or a bit worried, while 10 (9%) said they felt safe or not worried (Figure 8).

**Figure 9:**
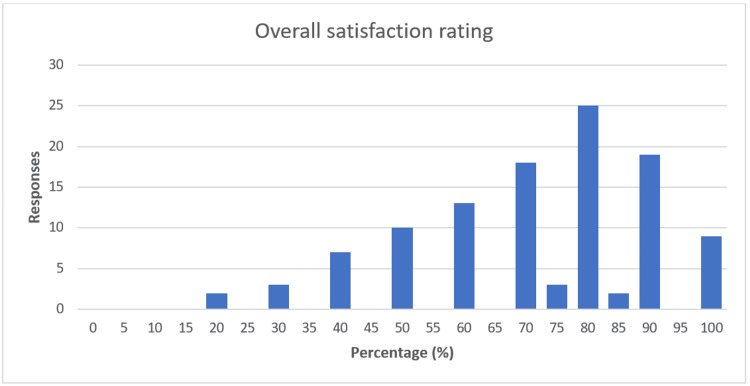
Bar chart showing the overall satisfaction rating for patients surveyed The X-axis shows the percentage of satisfaction; Y-axis shows the number of responses.

When asked if patients would come back to the clinic after they had attended and seen the COVID-19 precautions in place, 80 (72%) patients said they would attend again, while 31 (28%) said they would not. Patients were asked to rate their overall satisfaction with the communication process as a percentage. The average satisfaction rating was 71.7% (Figure 9).

Looking at how average patient satisfaction ratings compared when broken into age groups, patients aged <25 was 78.5%, 25-54-year-olds was 68.2%, 55-64-year-olds was 76.3%, and >64-year-olds was 71.1%. However, these differences were not statistically significant (p=0.22).

When separated into groups based on whether or not patients were out of the house during the day at work or school, out of the 71 patients who worked away from home or attended school, 73% said they would attend clinic again, and 27% said they would not. Of the 40 patients who were retired, unemployed, or working from home, 70% said they would attend the clinic again, while 30% said they would not.

## Discussion

Effective communication has many benefits to the overall surgical patient pathway pre-operatively. It improves patient understanding and reduces canceled appointments, ultimately saving NHS resources. There are many opportunities to improve communication with surgical patients from the point at which surgery is offered all the way through to the final post-operative follow-up appointment. The more a patient understands what is happening with their surgical treatment and plan, the more they will be willing to engage in advice and instructions both pre-operatively to optimize them for surgery and post-operatively to enable efficient rehabilitation, ultimately improving patient outcomes and patient satisfaction [14-16]. 

Many studies have looked at how different communication methods can improve these factors. Phone calls have been a mainstay of patient communication for many years, with great benefits. Studies have shown that phone calls play an important role in the pre-operative stage and significantly decrease surgery cancellation rates by allowing the healthcare provider to pass on instructions and the patient to ask questions during this period [17,18].

We now live in a world where more people than ever before own a mobile phone and have immediate access to communication with others. While phone calls remain the most popular form of communication with surgical patients' text messages have the benefits of being easily accessible, can be read at a convenient time for the patient, and will remain as a hard copy for future reference without requiring the patient to be held up speaking on the telephone at a time which may be inconvenient for them. Text messages have been shown to increase compliance with appointments and clinical protocols [19,20] as well as reduce anxiety levels before and after surgery both for patients and their family members [21]. There is also a big role that emails can play, especially amongst the younger population, as people have easy access to these on mobile phone devices.

Sawni et al. looked at communicating with adolescents in the USA in an area with a high level of poverty. They found that using cell phones was a very promising way to effectively communicate with that population through texting, phone calls/voicemail, email, and social media [22].

Another study by Wegrzyniak et al. in 2018 looked at the appointment reminder methods chosen by patients from the private orthodontic practice. The most common form of communication for their appointment reminder was email, followed by text messages, and lastly phone calls. The study looked at the rates of appointment no-shows, with texts having the lowest rate, followed by email; however, there was no significant difference between the methods, and all three had an average rate of 2.43% [23]. As Wegrzyniak's study was done before the pandemic, this suggests that patient preferences may have changed during the global pandemic, shifting more heavily towards phone call reminders as opposed to other electronic forms of communications.

A study looking at electronic communication in a vascular surgery clinic in February 2021 by Kelly et al. found that while phone calls are a popular method throughout all age groups, text messaging decreases in popularity as age increases [24]. This corresponds with the results of this study.

In terms of patient feelings toward healthcare during the COVID-19 pandemic, studies have found that good communication and planning by healthcare providers can increase how safe patients feel towards undergoing medical treatments [25,26].

Chen et al. looked at safety concerns of patients undergoing total knee replacement surgery and found that reassuring factors included pre-operative COVID-19 testing, use of personal protective equipment​​​​​​ (PPE) by hospital staff, speaking to their surgeon, and being reassured pre-operatively [27].

This study found that when patients were separated into groups based on whether or not they were out of the house during the day at work or school, those who worked away from home or attended school during the pandemic felt safer about COVID-19 and were more likely to attend clinic again, compared to those who were retired, unemployed, or working from home. This is consistent with other observations around the world. A large study by Ruiz-Frutos et al. looked at non-healthcare workers in Spain and found that among those who reported psychological distress during the pandemic, those who worked away from home felt safer than those who worked from home [28].

Breaking down safety ratings by age, the older patients felt less safe than the younger patients, and this was statistically significant. This could be due to the perceived higher risk of older patients to the COVID-19 virus.

This study is a follow-on from an audit done pre-pandemic in the same department by Ghunimat et al. [12]. One noticeable similarity between before and during the COVID-19 wave of infections is that preferred communication methods remain relatively varied, with all four methods having some patients to choose it. The main difference seems to be the shift towards phone calls becoming more popular, with it being the most popular by a small margin before the pandemic, to now being significantly more popular during the pandemic. This may be in part due to the well-documented isolation and loneliness that patients were feeling during lockdowns and isolations in the UK [29].

Another interesting difference between before and during COVID-19 is the preferred notice period for the patients' surgery. Before COVID-19, the majority of patients were happy with a notice period of one week, whereas, during the pandemic, the majority of patients wanted a notice period of between one and two weeks. This may indicate a shift in behaviors and beliefs caused by heightened anxiety levels, and patients feel they need more time to get ready and mentally prepare for their surgeries during a pandemic.

The COVID-19 pandemic has changed the way healthcare is provided, with many more consultations currently being done remotely than ever before. The way clinicians communicate with patients has changed and become more important as a result. The results from this study can be used to change clinical practice. In terms of communication with patients, sending more emails and text messages to younger patients while sending more letters to older patients can be trialed to optimize communication effectiveness while including phone calls for all patients' ages and genders.

In terms of aiming to keep patients safe during their appointments, in the department where this study was conducted, patients have been spread out more by opening up more clinics and aiming to reduce the numbers of patients in waiting areas at any one time. More work can be done to look at improving patient flow through patient-facing departments, as has been done throughout many hospital outpatient departments worldwide as a result of the COVID-19 pandemic [30].

This audit comprised data from a single surgical specialty and was performed at one hospital, which is a limitation of this study. Future work on this subject could aim to perform a multicentric study with a greater number of responses, spanning multiple medical and surgical specialties. This would allow further evaluation of how patients across other specialties prefer to be contacted.

## Conclusions

The COVID-19 pandemic has changed the way elective and outpatient healthcare is provided to patients. Healthcare organisations and departments need to continue adapting to ensure that patient safety is protected while maintaining efficiency. Communication preferences for trauma and orthopedic patients vary depending on several different factors including age and gender. All patients prefer phone calls, while younger patients also like texts and emails. Letters have good efficacy in the older surgical patient population as well as females. Therefore, it is important that the methods used for communication by healthcare staff are varied and adaptable to match patient demand with service provision and maximise safety and efficacy of patient care.

## Appendices

Patient Communication Preference Questionnaire

The impact of the COVID-19 pandemic has restricted face-to-face appointments with orthopedic healthcare professionals for orthopedic-related conditions. The Orthopaedic Team at Queen's Hospital Burton and Derby Hospitals Trust want to improve communication by using the most appropriate means of contacting patients peri-operatively. Currently, we are undertaking a study into the efficiency and patient satisfaction of our communication from a patient's perspective through this anonymous patient questionnaire. Thank you in advance for providing your views in this patient questionnaire.

How old are you: _____ years

What gender:  ____________                                                 

Please tick the options as appropriate

Q1) Are you currently working?

□ Yes (please continue to Q2) 

□ No (please continue to Q3)

Q2) During the COVID-19 pandemic where have you been based?

□ at home

□ working social distanced

□ in education at school

□ virtual learning from home

□ other, please specify: __________________________________

Q3a) What is your communication method preference (you can tick more than one answer)?

□ text

□ phone call

□ letter

□ email

□ other, please specify: __________________________________

Q3b) Do you have a preference? __________________________________

Q4) How many days' notice do you need before attending for clinic?

_______ days

Q5) Would you like confirmation by letter for clinic?

□ Yes 

□ No

Q6) How satisfied have you been with the communication to date?

0 _________________________________________________________ 100%

Q7) How much notice do you need before surgery?

_______ days

Q8) When you attended clinic how safe did you feel?

0 _________________________________________________________ 100%

Why?

__________________________________________________________________

__________________________________________________________________

__________________________________________________________________

__________________________________________________________________

Q9) Would you come to clinic again?

□ Yes

□ No
